# Evaluation of the surveillance system for invasive meningococcal disease (IMD) in the Netherlands, 2004–2016

**DOI:** 10.1186/s12879-019-4513-2

**Published:** 2019-10-17

**Authors:** Diederik A. H. Brandwagt, Arie van der Ende, Wilhelmina L. M. Ruijs, Hester E. de Melker, Mirjam J. Knol

**Affiliations:** 1 Centre for Infectious Disease Control, National Institute for Public Health and the Environment (RIVM), Postbus 1, 3720 BA Bilthoven, The Netherlands; 2 European Programme for Intervention Epidemiology Training (EPIET), European Centre for Disease Prevention and Control (ECDC), Gustav III:s Boulevard 40, 169 73 Solna, Stockholm, Sweden; 3 Public Health Service Utrecht region, Postbus 51, 3700 AB Zeist, the Netherlands; 4 Netherlands Reference Laboratory for Bacterial Meningitis (NRLBM), Amsterdam University Centers, Location Academic Medical Center, Meibergdreef 9, 1105 AZ Amsterdam, the Netherlands

**Keywords:** Evaluation, Surveillance, Meningococcal disease, Epidemiology, The Netherlands

## Abstract

**Background:**

Enhanced surveillance for confirmed cases of invasive meningococcal disease (IMD) was introduced in the Netherlands in 2003, in which reference laboratory data (NRLBM) are linked with notification data (OSIRIS). The quality of surveillance information is important for public health decision making. Our objective was to describe the system and evaluate it for data completeness and timeliness.

**Methods:**

Cases reported in the surveillance system from 2004 to 2016 were included. For the notification data, we used information on serogroup, vaccination status, mortality, and country of infection as indicators for record completeness. Notification times to regional and national level were calculated using the reported dates available in the notification database.

**Results:**

A total of 2123 cases were reported in the years 2004–2016, of which 1.968 (93%) were reported by the reference laboratory and 1.995 (94%) in the notification system. Of all cases, 1.840 cases (87%) were reported in both systems and could be linked. The serogroup was known in 86% of the notified cases, and was significantly higher (94%) in the years 2013–2016. Information on vaccination status, mortality and country of infection was available in 88, 99 and 97% of notified cases, respectively. Regional notification of cases occurred within one working day for 86% of cases and 98% were notified nationally within three days.

**Conclusions:**

A well performing IMD surveillance system was demonstrated and serogroup completeness has improved over the years. Underlining the need for reporting to both the clinical and laboratory surveillance system remains important to further improve the overall performance in supporting public health response and vaccination policy.

## Background

Invasive meningococcal disease (IMD) is caused by the Gram-negative bacterium *Neisseria meningitides* and can lead to serious manifestations including meningitis and septic shock. The case-fatality rate is high (in the Netherlands around 8%) and differs between the different clinical manifestations and age groups with a case fatality of up to 19% for septic shock. Severe long-term consequences are seen in 6% of all patients and include neurological problems like deafness and brain damage, leading to severe cognitive and functional problems, and can also include limb amputations in the case of septic shock [1].

Based on differences in the polysaccharide capsule, different serogroups of *N. meningitidis* are described. The main serogroups worldwide causing disease are serogroup A, B, C, W and Y; in Europe the most common serogroup is serogroup B [2]. Monovalent conjugate vaccines are available against serogroup C and quadrivalent conjugate or polysaccharides vaccines are available against a combination of serogroups A, C, W and Y. The available vaccines against serogroup B consist of multiple protein components (Bexsero) or two variants of one protein (Trumenba). In many European countries, vaccination against IMD is included in the national immunisation program [3].

**Fig. 1:**
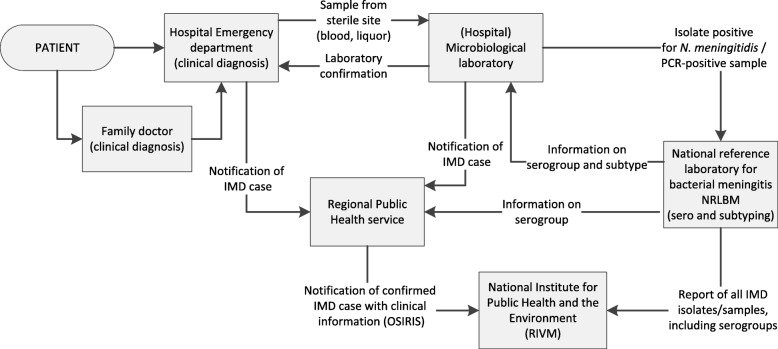
Data flow of the IMD surveillance system in the Netherlands since 2003

To be able to detect clusters or outbreaks and trends over time, and to evaluate vaccination programs, a good surveillance system including information on age, gender, serogroup, mortality and clinical presentation should be present. In the Netherlands, an enhanced surveillance system for IMD is in place, in which surveillance data from two sources are combined at the Dutch National Institute for Public Health and the Environment (RIVM) (Fig. 1).

The first data source is the electronic notification database at the RIVM, named OSIRIS, which was implemented in 2003. In the Netherlands, IMD is a notifiable disease since 1905 by law. According to the Dutch Public Health Act (WPG), both clinicians and laboratories should notify a confirmed case of IMD to a regional public health service (RPHS, in the Netherlands known as GGD) within one working day after diagnosis, in order to facilitate contact tracing and postexposure prophylaxis. If a case meets the notification criteria (Table 1), the RPHS reports the case within three working days to the national level (RIVM) for national surveillance. In addition to the EU notification criteria [4], the Dutch criteria also include information on the clinical picture. Cases that only meet the clinical or laboratory criteria should not be notified in the surveillance system. After receiving the notification, RPHSs actively collect additional demographic, epidemiological and clinical information of the patient. RPHSs also receive information on the serogroup through the NRLBM, in order to vaccinate risk contacts within 4 weeks since the date of onset of the index patient if the index patient has an infection with one of the serogroups of the ACWY-vaccine. The RPHSs report all additional information, including serogroup, with the notification in OSIRIS. All questions in the OSIRIS questionnaire have limited (multiple-choice) answer options or drop-down menus, for standardization of information. Furthermore, the questionnaires have an open-text variable for comments, which is used for communication between the RPHS and the RIVM when additional information is needed.

**Table 1:** Case definition for invasive meningococcal disease, used for notification to the national level in the Netherlands (OSIRIS)

*A person with at least one of the following symptoms:*	
*• Fever*	
*• Neck stiffness*	
*• Petechiae*	
*• Septic shock*	
*• Septic arthritis*	
*In combination with at least one of the following laboratory criteria:*	
*• Detection of N. meningitidis by culture of normally sterile material (*e.g. *blood or cerebrospinal fluid (CSF))*	
*• Detection of gram-negative diplococci in CSF, blood or biopsy of petechiae*	
*• Detection of DNA of N. meningitidis by PCR of normally sterile material (*e.g. *blood or CSF)*	

The second data source is the Netherlands Reference Laboratory for Bacterial Meningitis in Amsterdam (NRLBM, AMC/RIVM). In addition to the notification of a confirmed case to the RPHSs, clinical laboratories in the Netherlands submit to the NRLBM all invasive meningococcal isolates (from normally sterile sites) for serogrouping and further subtyping. Culture negative materials are submitted for PCR identification of meningococcus and serogrouping. Serogrouping is performed by using Ouchterlony gel diffusion or PCR, subtyping is done by sequencing. The NRLBM reports information on the serogroups and subtypes of IMD cases to the RIVM. Like the OSIRIS database, the information in this database is pseudonymized. The report contains some basic demographics, and information on the isolates like site of isolate, serogroup and subtype. At the RIVM, the information from the NRLBM is linked on individual case level with the notification data from the RPHSs. As there is no common identifier in both databases, the linking is done manually and based on basic demographics (year of birth, gender, 4-digit postal code) and by comparing date of diagnosis (OSIRIS) and date of sample (NRLBM). Since 2014, registrations are compared directly after receiving the report from the NRLBM. All cases before 2014 have been linked retrospectively. The aim of combining the two data sources is to have a complete overview of all IMD cases in the Netherlands, including epidemiological, clinical and microbiological data.

As no formal evaluation of the IMD surveillance system was performed since the implementation of OSIRIS in 2003, the objective of our study was to describe the representativeness of both data sources in the system and to evaluate the completeness of data and the timeliness of the system.

## Methods

### Study population

We used the actual dataset at the RIVM, in which the information from the NRLBM and the OSIRIS database was linked on case level. For analysis, we used information on all cases reported in the period from 1 January 2004 to 31 December 2016.

### Distribution of cases

All cases were categorized in three groups: cases that were reported in both databases, cases that were only reported in the NRLBM database and cases that were only reported in the OSIRIS database. We calculated the percentage of the total number of cases that is in one of the two systems. For cases that were reported in OSIRIS only, we manually checked the comment-variable for possible reasons why a case was not known in the NRLBM-database.

### Completeness

We used four variables as indicators for data completeness, as these variables were used since the implementation of OSIRIS in 2003: serogroup, vaccination status, mortality and probable country of infection. In OSIRIS these questions are answered by selection the right option in a drop down menu, with separate answer options for “no” and “unknown”. Variables were regarded as incomplete if the answer “unknown” was selected or no answer was selected. Completeness was calculated by dividing the number of cases with complete information, by the total number of cases in OSIRIS.

### Timeliness

To estimate the timeliness of the system, we calculated the time of notification to the RPHS (notification should be made within one working day) and the time of notification to the national level (RIVM) should be done in three days [5]. Time of notification to the RPHS was calculated as the difference between the date of diagnosis (manual entry) and the date that the notification was received by the RPHS (manual entry). Cases with a notification time of 2–4 days were manually corrected for bank holidays and weekend days. Cases with a (corrected) notification time of 0 or 1 day were defined as on time. Time of notification to the RIVM was calculated as the difference between the date of notification to the RPHS and the date of creating the record in OSIRIS (automated entry). Cases with a notification time of 4–6 days were corrected for bank holidays and weekend days. All cases with a (corrected) notification time of 0–3 days were defined as reported on time. Unusual high notification times (more than 150 days) and negative notification times were considered typing errors and therefore set as missing values. For timeliness, the percentages of cases reported on time and the mean and median notification times were calculated. For cases that were not notified on time, we assessed the comments for possible explanations of the delay. Based on the time variables in OSIRIS, we also described the mean and median time between day of onset and diagnosis, day of onset and day of notification to local level and day of onset and notification to national level, to illustrate the patient and diagnostic delay that should be taken into account for control measures.

### Statistical analysis

The distribution of cases over the two databases was calculated overall, and stratified by year, age categories and RPHS region. Data completeness and timeliness were calculated overall and stratified by year and RPHS region. We used chi-square tests to test for differences between the strata (StataCorp. 2015. *Stata Statistical Software: Release 14*. College Station, TX: StataCorp LP.).

## Results

### Distribution of cases

The complete database included 2123 unique cases of IMD within the studied period Jan 1st 2004 – Dec 31th 2016. Of these, the NRLBM reported 1968 cases (93%); 1995 cases (94%) were reported in the OSIRIS notification database. Of the 2123 unique cases, 1840 (87%) cases were reported in both databases and could be linked. One-hundred-twenty-eight (6%) cases were only reported in the NRLBM database and 155 (7%) cases only in the OSIRIS database.

Of 155 OSIRIS only cases, in 86 cases (67%) were diagnosed based on PCR without confirmation by positive culture and therefore no isolate was submitted to the NRLBM. For an additional 15 cases (11%) the diagnosis was based on microscopy only and also no isolate was available. In 12 cases (9%) typing of the isolate was performed in another laboratory (mainly in other countries). For 16 cases (12%) it was commented that typing was performed by the NRLBM, but none of the NRLBM-only cases matched with these OSIRIS-only cases.

**Fig. 2:**
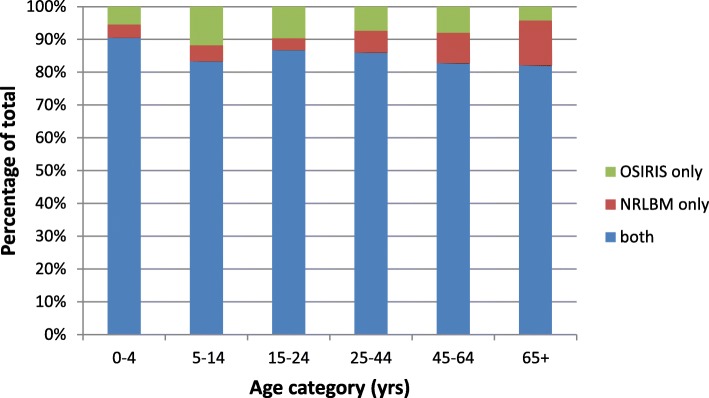
Relative distribution of cases reported in both or one of the databases per age category, IMD cases in the Netherlands, by age category, 2004–2016 (*N* = 2123)

The percentage of cases that was reported in both databases, ranged per year from 81% (2014) to 91% (2015), but was not significantly different between the years (*p*-value = 0.8). The distribution of cases over the databases was significantly different for the different age categories (*p* < 0.001). The percentage of cases that were reported in both systems varied from 82% for the group of 65 years and older to 91% for the 0 to 4 year olds. The highest rate of cases that was only reported by the OSIRIS database was seen in the 5 to 14 year olds (12%), the highest percentage that was only reported in the NRLBM database was seen in the group of 65 years and older (14%) (Fig. 2).

For 2107 unique cases (99%), the municipality of residence was available in the databases. All 16 cases without information on the place of residence were reported in the NRLBM database only. On RPHC level, the percentage of cases reported in both systems varied from 76% in the region with the lowest proportion, to 98% in the region with the highest proportion (*p* < 0.001).

### Data completeness in OSIRIS-database

Of 1995 cases in the OSIRIS-database, 1708 (86%) were reported with information on the serogroup. This percentage was higher among the 1840 cases that were linked with the NRLBM-database, with 1671 (91%) cases with information on the serogroup. The percentage with a known serogroup varied significantly (*p* > 0.001) over the years, ranging from 74% (2008) to 95% (2013 and 2015). The percentage of cases with information has increased significantly over the years, from 80% in the years in 2004–2008, to 94% in the years 2013–2016 (*p* < 0.001). Per RPHC, the percentage differed significantly, ranging from 67 to 94% (p > 0.001). In the years 2013–2016, nine of the 25 RPHS regions had 100% completeness for the serogroup information.

Vaccination status was reported in 1760 (88%) of the cases. This percentage ranged from 82% (2013) to 91% (2005) over the years (*p* = 0.55). The completeness showed a decreasing trend from the years 2004–2008 (90%) to 2013–2016 (86%) but this was not significant (*p* = 0.07). Per RPHC region the percentages were significantly different, ranging from 63 to 97% (*p* < 0.001). Four from the 25 RPHSs had 100% completeness for vaccination status in 2013–2016.

Information on mortality was entered for 1972 (99%) of the cases. This percentage was not significantly different between the years (*p* = 0.21) and varied from 97% (2013) to 100% (several years). On RPHC region level the completeness varied from 86 to 100% (*p* = 0.001), although the RPHC with 86% completeness was the only RPHC below 96%. Twelve RPHCs had 100% completeness for information on mortality. The overall case fatality rate was 6% and varied from 3 to 9% in the different years (*p* = 0.32).

The probable country of infection was entered for 1.934 (97%) cases, based on the questions “most probable country of infection” and “other possible countries of infection”. This percentage also did not vary significantly between the years (*p* = 0.18), with values ranging from 94% (2014) to 99% (2012). On RPHC level, the completeness varied significantly from 87 to 100% (*p* = 0.01), with four RPHSs with a completeness of 100%. The RPHC with 87% completeness, was the only RPHS with a completeness below 92%. For most patients the probable country of infection was the Netherlands (1885, 95%); only eight patients (0.4%) contracted the disease outside Europe.

**Fig. 3:**
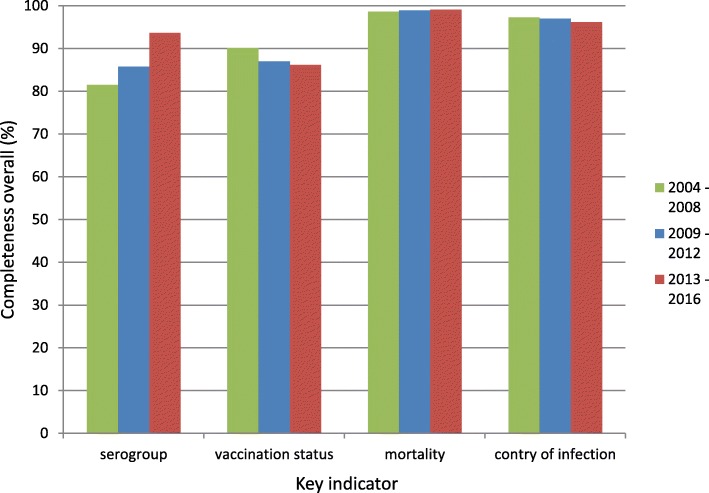
Data completeness for the four key indicators, stratified by time period. Confirmed IMD-cases in the OSIRIS database, the Netherlands, 2004–2016 (*N* = 1995)

A graphical summary of the data completeness over the three different time periods (2004–2008, 2009–2012 and 2013–2016) is shown in Fig. 3.

### Timeliness

For 1953 (98%) OSIRIS cases, the time between diagnosis and notification to the RPHS could be calculated. One-hundred-thirty-five (7%) entries with negative and unusually high numbers were defined as missing values, leaving 1818 entries for analysis. Of these cases, 1564 (86%) were notified within one working day. The average notification time was 2.3 days (median 0 days; IQR 1 day). Of the 254 cases that were not notified on time, for 65 cases (26%) a possible explanation was reported in the comment field. Of these, 23 cases were accidently not notified by the hospital, 16 cases were notified with a delay due to late laboratory results or due to human or technical error. In 12 cases no notification was made initially as all laboratory tests remained negative and in 9 cases it was unclear whether the clinical picture matched the notification criteria. The remaining five cases were diagnosed in another country. The timeliness improved significantly over the years from 83% in the period 2004–2008 to 93% in the years since 2013 (*p* < 0.001). Per RPHS region, the timeliness varied from 64 to 96% (*p* < 0.001). Thirteen RPHS regions had 100% timeliness in the years since 2013. Overall, 99% of the cases were notified to the RPHS within one week after diagnosis since 2013.

The notification time to the national level could be calculated for all 1995 OSIRIS cases; 1982 cases were used for analysis after excluding negative or unusually high numbers. Of these, 1928 (98%) cases were notified within 3 days to the national level. Per year the timeliness varied from 96 to 100% (*p* = 0.45). The timeliness did not differ significantly between the RPHSs (range 92 to 100%, *p* = 0.15). On average, the RPHSs notified cases to the national level in 0.8 days (median 0 days; IQR 0 days). Based on the information on the date of onset reported in OSIRIS, the median time between date of onset and date of confirmation was 3 days (IQR 3 days). Median time between date of onset and notification to local level was 3 days (IQR 3 days) and median time between date of onset and notification to national level was 4 days (IQR 4 days).

## Discussion

In our study, we described a comprehensive surveillance system including demographic, epidemiological, microbiological and clinical data. Most cases were reported in both databases with only small percentages of cases missing in either of the data sources. The completeness of the data in the notification database is high, and a high percentage of cases are notified in time, which facilitates control measures in an early stage. For data completeness, there were some significant differences at RPHS level, with individual RPHSs with a much lower completeness scores than the others. However, there was no consistent pattern regarding the best and lowest score per indicator.

For a complete evaluation of a surveillance system, it is recommended to also include more qualitative indicators like usefulness, simplicity, flexibility and acceptability [6]. As the main objective of our study was to describe the proportion of cases reported in both systems and the quality of the data in the national surveillance system, we focused on existing data available in the national surveillance databases and therefore only on the quantitative quality indicators.

For 87% of all unique records, linking between the two sources was possible. As no common identifier was available in both databases, the linking was done probabilistically, based on basic demographics and (approximate) date of diagnosis. Manual linking is very labor-intensive, especially in a period with high numbers of reported cases and is probably also less accurate compared with using a common unique identifier (deterministic linking). Introducing a common identifier could therefore facilitate the linking process at the RIVM.

For 73% of the OSIRIS only cases, it was clear from comments that no isolate was sent to the NRLBM for serogrouping, or serogrouping was done in another hospital, so these would still have been missing with deterministic linking. For the 128 NRLBM only cases, the exact reasons for not reporting in OSIRIS are unknown. It is possible that the patient did not comply with the clinical notification criteria, although it is hard to imagine a patient with *N. meningitidis* isolated from a normally sterile site without having clinical symptoms like fever. Another explanation could be a failure (technical or human) by the regional laboratories to notify a case to the RPHS. Based on the comments in OSIRIS, in 28 cases (1.4% of all OSIRIS cases) no notification was made by the regional laboratory to the RPHS, before the RIVM contacted the RPHS. A third explanation could be a failure at the RPHS level to notify a case in OSIRIS. Since recently the RIVM contacts the RPHSs actively when a case with known residence is reported by the NRLBM, without a matching case in OSIRIS, to get better insight into reasons for not reporting in OSIRIS.

The 87% of cases that is reported in both databases is high compared with some comparable studies from other European countries with similar IMD surveillance systems. In an Irish study, looking at the surveillance data from 1999 to 2015, for 83% of all unique records a link could be made between the disease reporting system and the laboratory surveillance system [7]. From the disease reporting database, 87% of the cases were also reported in the laboratory database, from the laboratory database 94% of the cases were also reported in the disease reporting database. In a German study, looking at the year 2003 only, the percentage of linked cases was 61% [8]. In this study, the number of cases in the disease reporting system was much higher than in the laboratory surveillance system. Based on a two-source capture-recapture analysis in this study, the sensitivity of the disease reporting system was high with 90%. In our study, we decided not to perform a capture-recapture analysis, as the two sources are not independent; the isolate positive for meningococcus at the regional laboratory, is the starting point for both the notification to the RPHS as sending in material to the NRLBM.

The data completeness of OSIRIS cases is high, with serogroup information entered in 87% of the cases. When we limit the analysis to cases that are reported in the NRLBM database, this percentage increases to 91%. When we further restrict to recent cases (2013 and later), for 98% of the cases that are reported in both databases, information on the serogroup is reported. The high percentage in serogroup completeness is also the result of policy of the RIVM, in which the RIVM actively requests to enter the serogroup information, when a notification is sent for finalization in OSIRIS. Since 2006, for 38% of all OSIRIS cases, such a request has been made. The percentage of requests per year was not significantly different between the years.

For vaccination status, the completeness of data is high, although the improvement we have seen for serogroup in recent years is not seen for this variable. We expect the validity of the information entered by the RPHSs is high, as these request the vaccination status (type of vaccine and date of vaccination) of each case from a regional office of the national vaccination registry in most cases. To monitor the effect of the new vaccination schedule, it is important to report the vaccination status, especially when the serogroup of a case is known. With recent media attention for the increasing numbers of IMD-cases caused by serogroup W (IMDW) in the Netherlands and the new vaccination schedule and therefore increasing awareness for IMD, we expect the completeness of this variable will increase in the next years. Furthermore, as the OSIRIS questionnaire has been updated because of the new vaccination policy, the question has been brought under attention of public health professionals again.

Data completeness for country of infection and mortality have both been high since the implementation of OSIRIS in 2003. The decreasing number of deaths for IMD since 2004 is also in line with the official death records (based on ICD10 code: A39), but the numbers are slightly higher in OSIRIS [9]. In the official death records, the higher number of deaths in 2016 in ORISIS (13 deaths, of which 7 in IMDW cases) is not seen, which might be the result of official death records that are finalized before the diagnosis IMD is confirmed. The true number of deaths due to IMD might also be higher than reported in OSIRIS, as some of the cases might die after the finalization of the record in OSIRIS. With the higher mortality among IMDW cases shown in the UK (12%) and in the Netherlands (17%), the question on mortality remains important [10, 11]. In light of the more non-specific presentation of cases with IMDW [12, 13], it might be useful to focus more on clinical symptoms. Through the years, questions on clinical manifestations have varied a lot and have also been absent for many years before reintroduced using drop down answer menus with standardized and more detailed formats in 2015. The completeness for the clinical manifestation variables in the years 2015 and 2016 was 100%. Since 2017, detailed questions on disease symptoms, including gastro-intestinal symptoms, are included in the questionnaire as well. Information on sequelae is not included in the OSIRIS-database as the questions on clinical symptoms focus on the symptoms on the day of onset. The main task of the RPHSs is to implement control measures as early as possible, for which a detailed follow-up on one single patient is not needed. Therefore the follow-up period on cases is short and case records are finalized early in OSIRIS.

Timely notification is important to enable the RPHS to provide post exposure prophylaxis to close contacts and to deal with unrest among close contacts in case of severe illness or death. The timeliness of notifications to the RPHS and from RPHS to the national level was good, with 86 and 98% notified on time respectively, with a significantly higher percentage in recent years for the notification to RPHSs. One major limitation in the calculation of timeliness is the entry of dates. Only the date of notification to the national level is entered automatically, by creating the notification in OSIRIS. All other date values are entered manually by the RPHS, which is prone to typing errors. For example, for 131 cases, we calculated a negative time between diagnosis and notification. Furthermore, there is no clear definition of the day of diagnosis (probable clinical diagnosis or lab confirmation) and the day of notification to the RPHS. Some RPHSs use the day mentioned on the lab result, other RPHSs use the day the lab result has arrived or been processed. In the latter case, reporting delay from diagnosis to notification might be missed. In practice, the timeliness of control measures (chemoprophylaxis and vaccination) is more important than the timeliness of notification. The RPHSs report their control measures only in their own case management systems. As all RPHSs in the Netherlands are separate organizations, the data from these case management systems are not available to others. Therefore we have no insight in the timeliness of the control measures on regional level.

For all of the four key indicators for completeness as well as for the timeliness, some individual RPHSs had a significantly lower score than others. For the four completeness indicators, it was not the same RPHS scoring lowest. When stratifying for the three time periods also different RPHSs scored lowest per time period per indicator. The lower completeness seems therefore not a structural problem, but still we think it remains important to pay attention on data completeness. The results of our completeness analysis will therefore be shared with the individual RPHSs. For timeliness, already a system is in place, in which the RIVM sends a report to RPHSs with information on their timeliness of all notified cases in OSIRIS. This helps RPHSs to monitor trends over time and to investigate structural problems in their notification procedures [14].

## Conclusions

The surveillance system for IMD in the Netherlands is performing very well, with high completeness and timeliness, but there is some room for improvement. The linking of cases is currently done manually, which is labor-intensive. With an increasing number of cases, the manual probabilistic linkage can become more difficult, as there is a chance more cases will have similar demographic characteristics, especially in case of local outbreaks. Therefore, we could consider the implementation of a common unique linking variable, which facilitates automated (deterministic) linking and is less vulnerable for data entry errors. This linking should be in line with the current privacy laws in the Netherlands and Europe. The reporting of serogroup information in OSIRIS becomes also less relevant when the two data sources are combined automatically. The entry of this information can be removed from the questionnaire, making the notification less time-consuming for the RPHSs. Another improvement of the system would be to further promote regional laboratories to notify timely to the RPHSs and to send in materials to the NRLBM, even when there is no positive culture. With new molecular techniques, it is often possible to still determine the serogroup of a case. The improvements will help to increase the number of cases with full information on microbiological, epidemiological and clinical data and bring the number of cases in the surveillance system closer to the real number of cases. This will increase the performance of the surveillance system in detecting clusters and trends over time, which might lead to new opportunities for control measures.

## Data Availability

The data used were collected through the routine surveillance system of IMD in the Netherlands, but restrictions apply to the availability of these data, and are not publicly available. Data are however available from the authors upon reasonable request and with permission of the Centre for infectious disease control or the RIVM, Bilthoven, the Netherlands. Two of the authors (MK and AE) are responsible for the surveillance of IMD in the Netherlands and have therefore access to the raw data.

## Abbreviations

CSF: Cerebrospinal fluid
IMD: Invasive meningococcal disease
IMDW: Invasive meningococcal disease caused by serogroup W
IQR: Interquartile range
NRLBM: Netherlands Reference Laboratory for Bacterial Meningitis
OSIRIS: National electronic notification database (based at RIVM)
PCR: Polymerase chain reaction
RIVM: Dutch National Institute for Public Health and the Environment
RPHS: Regional Public Health Service
WPG: Dutch public health act [in Dutch: Wet Publieke Gezondheid]
